# Metal-doped carbon dots for biomedical applications: From design to implementation

**DOI:** 10.1016/j.heliyon.2024.e32133

**Published:** 2024-05-31

**Authors:** Jin Qi, Pengfei Zhang, Tong Zhang, Ran Zhang, Qingmei Zhang, Jue Wang, Mingrui Zong, Yajuan Gong, Xiaoming Liu, Xiuping Wu, Bing Li

**Affiliations:** a Shanxi Medical University School and Hospital of Stomatology, Taiyuan, 030001, Shanxi, China; b Shanxi Province Key Laboratory of Oral Diseases Prevention and New Materials, Taiyuan, 030001 Shanxi, China; c Taiyuan University of Science and Technology, Taiyuan, 030024, Shanxi, China; d The First Hospital of Shanxi Medical University, Taiyuan, 030001, Shanxi, China

## Abstract

Carbon dots (CDs), as a new kind of fluorescent nanomaterials, show great potential for application in several fields due to their unique nano-size effect, easy surface functionalization, controllable photoluminescence, and excellent biocompatibility. Conventional preparation methods for CDs typically involve top-down and bottom-up approaches. Doping is a major step forward in CDs design methodology. Chemical doping includes both non-metal and metal doping, in which non-metal doping is an effective strategy for modulating the fluorescence properties of CDs and improving photocatalytic performance in several areas. In recent years, Metal-doped CDs have aroused the interest of academics as a promising nano-doping technique. This approach has led to improvements in the physicochemical and optical properties of CDs by altering their electron density distribution and bandgap capacity. Additionally, the issues of metal toxicity and utilization have been addressed to a large extent. In this review, we categorize metals into two major groups: transition group metals and rare-earth group metals, and an overview of recent advances in biomedical applications of these two categories, respectively. Meanwhile, the prospects and the challenges of metal-doped CDs for biomedical applications are reviewed and concluded. The aim of this paper is to break through the existing deficiencies of metal-doped CDs and fully exploit their potential. I believe that this review will broaden the insight into the synthesis and biomedical applications of metal-doped CDs.

## Graphical abstract

**Figure:**
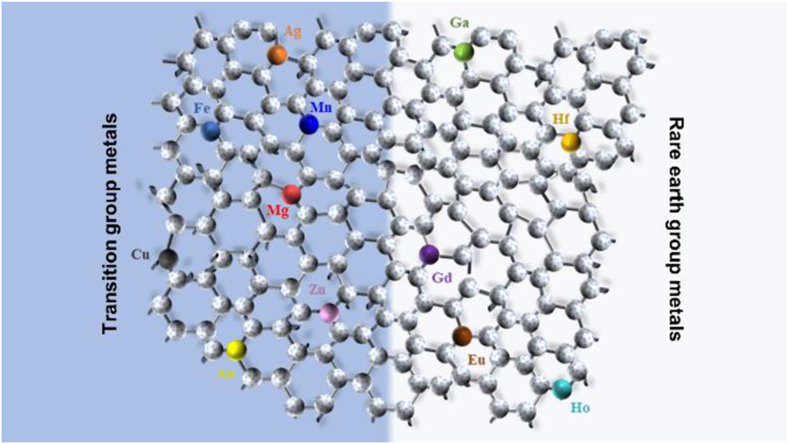

## 1 Introduction

**Fig. 1:**
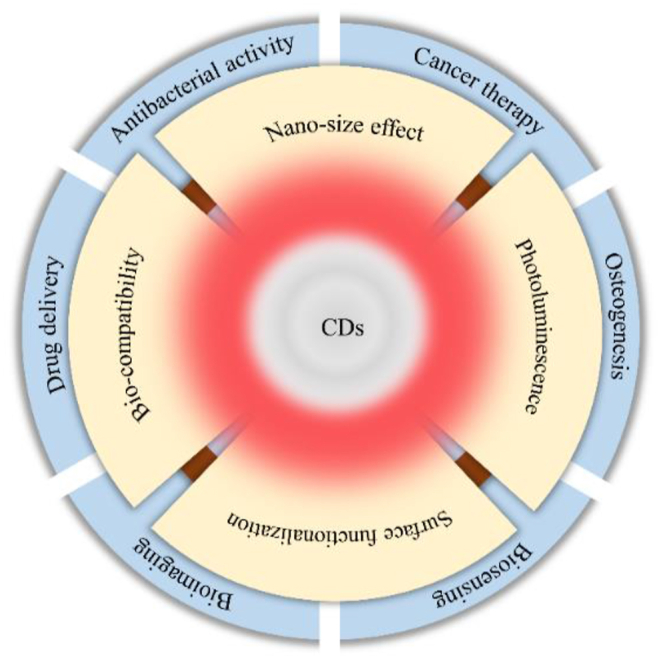
Properties and biomedical applications of CDs.

In recent years, as new members of the carbon nanomaterial family, CDs have attracted a tremendous amount of interest from researchers. CDs are usually defined as spherical nanoparticles with dimensions less than 10 nm, and in general, the structure of CDs consists of a carbon core and oxygen/nitrogen-containing surface groups. The carbon core structure usually consists of sp^2^ and sp^3^ carbon atoms, while the surface consists of a number of common functional groups such as amino, epoxy, carbonyl, aldehyde, hydroxyl and carboxylic acids [1,2]. CDs have shown great potential in biomedical applications due to their properties such as nanoscale size effect of less than 10 nm, good bio-compatibility, superior surface functionalization and outstanding photoluminescence properties [[3], [4], [5]]. For example, fluorescent probes as detection substances [[6], [7], [8]],transport carriers for drug delivery [9,10], bioimaging agents for in vivo tracing, etc. (Fig. 1) [11,12]. Since the serendipitous discovery of fluorescent nanoparticles in Electrophoretic purification purified single carbon nanotubes by Xu et al., in 2004, a number of methods have been proposed for the synthesis of CDs [13]. Depending on the carbon source, there are generally two types of methods: top-down and bottom-up. Top-down is the breaking down of bulk carbon materials such as graphene and carbon nanotubes into nanoscale structures through arc discharge, ultrasound, laser ablation, and electrochemical oxidation, which can be used for mass production. However, these methods have strict reaction conditions, limited choice of raw materials, tend to introduce impurities, synthesized materials are not homogeneous in size, and take a long time to achieve the desired results. Whereas bottom up synthetic classification is a simpler, economical and easier way to synthesize CDs by hydrothermal methods, microwave-assisted techniques, acid oxidation, carbonation and pyrolysis. Among them, hydrothermal/solvothermal method is the most ideal method to prepare CDs. The hydrothermal method involves placing the carbon source solution in an autoclave oven and heating it for several hours at the appropriate reaction temperature. The solvothermal method is similar to the hydrothermal method in that an organic solvent is used instead of water. Notably, this method has been successfully used for large-scale preparation of CDs and could be used for industrial-scale CDs preparation if the preparation time and energy consumption can be effectively reduced. Microwave-assisted technology involves irradiating the reaction medium with microwaves in a microwave oven to generate extremely high temperatures in a matter of minutes. The pyrolysis method has a desirable photoluminescence quantum yield (PLQY) and simple operation, but the dimensional inhomogeneity and susceptibility to agglomeration of the resulting CDs severely reduces their enzyme-like activity, limiting their potential for catalytic applications. In recent years, emerging technologies such as plasma, magnetothermia, and microfluidics have been used to produce CDs in batches ranging from a few grams to more than a hundred grams per batch. Advantages of these strategies include short reaction times, high productivity, and low energy consumption [[14], [15], [16]].Nevertheless, the applications of CDs are limited by the optical property drawbacks associated with conventional preparation methods, which include poor fluorescence quantum yield (QY), low visible light usage, and short emission wavelengths. Theoretically, CDs have the general properties of carbon materials, such as flaws and doping potential. Besides that, they show many special properties such as quantum confinement effects and surface modification [17]. The fluorescence of CDs essentially stems from the quantum confinement of the sp^2^ carbon building core. Taking advantage of the above properties, it has been demonstrated that introducing heteroatoms to alter the overall electronic distribution and associated electronic energy levels of CDs is an effective way to modulate the CDs' fluorescence and other characteristics [18]. In order to overcome the defects caused by the traditional preparation methods, its size, morphology and structure are modified by heteroatom doping, which of the CDs, thereby expanding the application scope in the field of biomedicine.

**Fig. 2:**
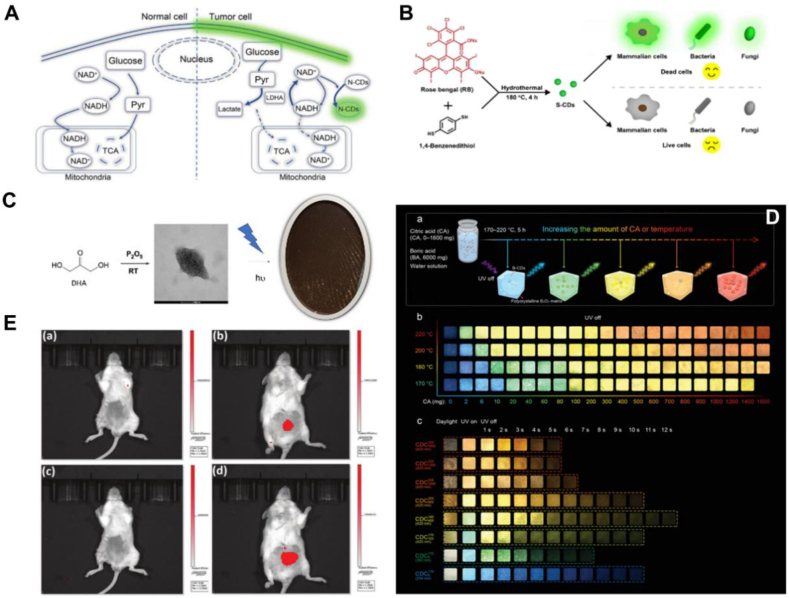
Application of Non-metallic doped CDs (A) N-CDs provide new therapeutic ideas for early prevention of tumors. Reprinted with permission from Li et al. (B)S-CD offers bright applications in cell imaging and cell viability assessment. Reprinted with permission from Wang et al. (C) P-CDs are potential alternatives for fingerprint imaging and security screening needs. Reprinted with permission from Manuel et al. (D) B-CD has potential applications in multidimensional information encoding and anti-counterfeiting. Reprinted with permission from Ding et al. (E) N-CDs-F offers new perspectives in the application of in vivo NIR imaging. Reprinted with permission from Jiang et al.

It has been shown that CDs have an electronic structure, exhibiting s/s*, s/p*, p/p*, n/p* and n/s* electronic transitions, which are closely related to their physicochemical properties, especially their optical properties (absorption and fluorescence) [19].Chemical doping is an effective method to modify the basic properties of CDs, which can effectively regulate the structure and electron distribution of CDs [20]. Non-metal doping and metal doping are the two categories of doping. Non-metal doping includes nitrogen (N), sulfur (S), phosphorus (P), boron (B), fluorine (F) and other doping element. Among them, nitrogen doping has been more widely studied and has been shown to be the most obvious strategy to enhance the properties of CDs [21]. The two types of metal doping are called transition group metal doping and rare-earth group metal doping, in which the transition group metal including iron (Fe), silver (Ag), copper (Cu), zinc (Zn), magnesium (Mn), nickel (Ni), etc., and the rare-earth group metal including lanthanide elements such as gadolinium (Gd), europium (Eu), hafnium (Hf) and so on. Non-metal doped CDs have been demonstrated as an effective strategy in adjusting the fluorescence properties of CDs and improving the photocatalytic performance in several fields. Li et al. successfully prepared nitrogen-doped carbon dots (N-CDs) by hydrothermal method using 2,3-diaminophenazine as raw material. This work reveals that the occurrence of fluorescence-enhanced aerobic glycolysis based on NAD-induced emission of N-CDs can effectively distinguish between tumor cells and normal cells, and fluorescent labelling of cells with aberrant energy metabolism to give an early warning of tumor development, which provides a new therapeutic idea for the early prevention of tumors in the clinical setting [22]. Wang et al. used rose bengal and 1,4-dimercaptobenzene as raw materials to prepare ultra-small (∼1.6 nm), ultra-bright (PLQY:∼78 %), excitation wavelength-independent sulfur-doped carbon dots (S-CDs), which could rapidly pass through the surface of dead cells to enter into the interior of dead cells but not live cells, verifying that S-CD can rapidly (∼5min) and accurately distinguish between dead and living cells in almost all cell types, and has better bio-compatibility and higher photostability than commercial live/dead staining dyes, ensuring its promising application in cell imaging and cell viability assessment [23]. Manuel et al. prepared a nanopowder of phosphorus-doped CQDs (P-CDs) with bright orange fluorescence under UV light using 1,3-dihydroxyacetone and phosphorus pentoxide. This work demonstrated the robustness of P-CDs for fingerprint imaging, which is a promising alternative for potential applications in security screening needs [24]. Ding et al. prepared boron doped carbon dots (B-CDs) with continuously tailorable, panchromatic, long-lived RTPs in the range of 466–638 nm by pyrolysis of precursors with different mass ratios of citric acids and boronic acids at different temperatures. This leads to the formation of luminescent B-CD centres in a rigid polycrystalline B2O3 matrix, which effectively stabilizes the triplet excited state of B-CD. As a result, the composite material changes to a phosphorescent material over a relatively long period of time (5–12s) after removal of the radiation source. This work explores the potential application of B-CD for multidimensional information encoding and anti-counterfeiting applications [25]. Jiang successfully prepared a novel D-π-conjugated N-CDs-F using urea and NH4F as dopants. Due to the formation of a special D-π-A conjugated structure, N-CDs-F not only exhibits strong absorption in the full spectrum of UV–vis–NIR, but also shows excellent two-, three- and four-photon excitation upconversion fluorescence, and shows bright deep red to near-infrared fluorescence both in vitro and in vivo, which can be effectively used for in vivo NIR imaging. This work opens up a new avenue for the practical application of CDs in the clinic (Fig. 2) [26]. In recent years, metal-doped CDs have received much attention as an emerging doping technology. Compared to non-metal doping, metal atoms are better electron donors with more unoccupied orbitals and larger atomic radius. Introducing metal ions into CDs will provide more opportunities to modulate the charge density and form of charge transfer between CDs and metal ions, thus improving the physicochemical properties of CDs [18,27]. After surface passivation or functionalization, the surface defects become more stable, facilitating effective radiative complexation of surface electron confinement, thus displaying enhanced optical properties [28]. Metal-doped CDs exhibit superior sensing for some small molecules based on a unique intra-particle Förster resonance energy transfer (IPFRET) system [29]. The introduction of metal ions into CDs affected the spin density and charge distribution and increased the number of catalytically active centre in CDs, thereby enhancing the catalytic performance [30].

Currently, there are more reviews published on non-metal doped CDs and few review articles on metal doped CDs. This paper innovatively divides metals into two major categories: transition group metals and rare-earth group metals. First, using several examples, we provide a summary and analysis of the most recent advancements of transition group metals in biological fields including antibacterial activity, cancer therapy, osteogenesis, biosensing and bioimaging, etc. Then we provide a brief overview of the latest developments of rare-earth group metals in the biomedical application. Finally, based on the existing work, an outlook analysis is presented highlighting the prospects and challenges of metal-doped CDs in biological applications.

## 2 Biomedical applications of transition group metal-doped CDs

The term transition element was first introduced by Mendeleev and refers to a series of metallic elements in the d and ds regions of the periodic table. Transition metals are cofactors required for many life-critical proteins and are essential for all life forms [31]. Transition group metal-doped CDs exhibit distinct optical properties and physicochemical properties with significant advantages over non-doped CDs. Examples include larger Stokes shifts to avoid self-absorption/energy transfer, longer excited state lifetimes, wider spectral windows and improved chemical and thermal stability [32]. In the following, we will through a large number of examples provide a comprehensive overview and analysis of the latest biomedical uses of transition group metal-doped CDs in biomedical fields including antibacterial activity, cancer therapy, osteogenesis, biosensing and bioimaging.

### 2.1 Antibacterial activity

Since Fleming's discovery of penicillin in 1928, the invention of antibiotics has revolutionized the field of medicine, saving billions of people from the threat of bacterial infections [33]. However, bacterial antibiotic resistance has emerged as a result of overuse of antibiotics and the continuous evolution of bacteria. As an emerging global crisis, drug-resistant bacteria are developing rapidly around the world, and the emergence of "superbugs" is increasing the mortality rate from disease and posing a serious threat to human life and health, making bacterial infections a huge public health risk [34]. It is predicted that bacterial infections will kill 10 million people a year and cost $100 trillion to treat after 2050 if without action is taken to reduce bacterial resistance or develop new antibiotics. At the same time, the number of deaths will surpass the number of deaths caused by cancer, which is currently the deadliest disease globally [35]. The development of new antibiotics is slow and they are only effective antibacterial agents in the short term, and relying solely on antibiotics to treat bacterial infections will become increasingly challenging and will not address the root causes of bacterial resistance [36]. With the gradual improvement of living standards and the significant increase of health awareness, human beings are more concerned about the environment in which they live. In order to effectively prevent various pathogenic bacteria in the environment from adversely affecting human health, the development of excellent antibacterial material systems to replace traditional antibiotic therapy is of great significance to national health and safety in the context of the new era. As a new member of the nanomaterial family, CDs utilize multiple antibacterial mechanisms to exert their antibacterial properties, greatly avoiding the challenge of bacterial resistance in clinical treatment. Transition metals have unfilled valence d-orbitals, the metal doping provides more opportunities to modulate the charge density and form of charge transfer between CDs and metal ions, which improves the physicochemical properties and antibacterial properties of CDs, and exerting great antibacterial potential [37].

**Fig. 3:**
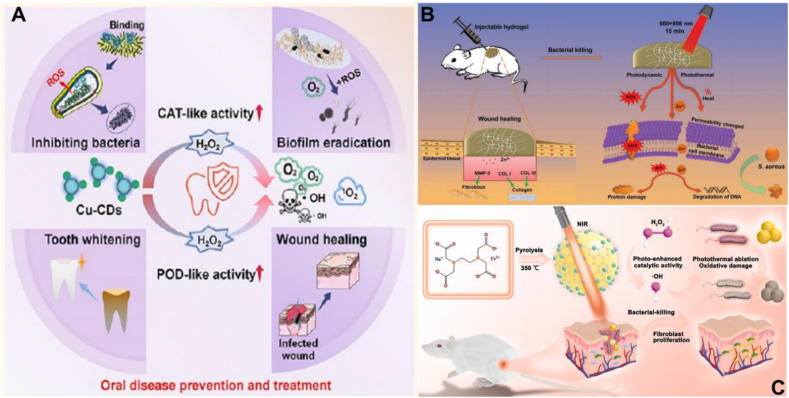
Antibacterial applications of transition group metal-doped CDs (A) Cu-CDs have great potential for improving the human oral microenvironment, preventing oral infectious diseases and tooth staining. Reprinted with permission from Liu et al. (B) Synthesis of DFT-C/ZnO hydrogels using C/ZnO to achieve efficient bactericidal ability in a short period of time and long-term prevention of bacterial infections. Reprinted with permission from Xiang et al. (C) Fe-CDs exhibit superior antibacterial properties and therapeutic effects in promoting wound healing under NIR light irradiation. Reprinted with permission from Liu et al.

Liu et al. designed and synthesized copper-doped carbon dots (Cu-CDs) with alternative bactericidal, biofilm eradication, wound healing and whitening activities at optimal temperatures. Their excellent catalase-like (CAT-like) and peroxidase-like (POD-like) activities, which significantly increased ROS and O_2_ production in the oral cavity, provide a theoretical basis for a range of therapeutic treatments. Notably, Cu-CDs have strong binding to peptidoglycan (PGN) and lipopolysaccharide (LPS) force, which ensures effective capture of bacterial cells and higher local dose, and thus they show excellent antibacterial ability against *E. coli* and *S. aureus.* The work synthesized a nano-mouthwash with very promising clinical applications, which has great potential for improving the microenvironment of the human oral cavity and preventing oral infectious diseases and tooth staining (Fig. 3A) [38]. Xiang et al. successfully synthesized DFT-C/ZnO hydrogels by selected carbon quantum dot (CQD)-modified ZnO (C/ZnO) composites as functional NPs. Rapid assembly of dopamine (DA) and folic acid (FA) by cross-linking of transition metal ions. Due to the synergistic effect of the released Zn^2+^ with the photothermal and photodynamic effects of the embedded NP, the DFT-C/ZnO hydrogel not only achieves long-term prevention of bacterial infections, but also possesses an efficient bacterial killing ability in a short period of time. In addition, Zn^2+^ is effective in promoting fibroblast growth, thereby improving tissue growth during wound healing. This study will provide a new insight into the design of hydrogels based on natural materials with multiple biological functions (Fig. 3B) [39]. Liu et al. developed ultrasmall Fe-doped carbon dots (Fe-CDs,3 nm) with excellent photothermal conversion and photo-enhanced enzyme-like properties by a simple one-pot pyrolysis method using NaFeEDTA. The combination of surface defects in the CDs and Fe-doping will lead to a highly stable and dense distribution of active sites with improved catalytic efficiency compared to undoped CDs. Fe-doping endowed the CDs with light-enhanced peroxidase (POD)-like activity, and they also exhibited excellent photothermal conversion properties and excellent photostability under near-infrared laser irradiation, which enabled the Fe-CDs to generate thermal and reactive oxygen species (ROS) for killing Gram-positive and Gram-negative bacteria. The antibacterial rates reached 99.68 % and 99.85 % against *S.aureus* and *E.coli*, respectively. In addition, Fe-doping and NIR laser irradiation contributed to fibroblast proliferation, neovascularization and collagen deposition, resulting in the therapeutic effect of Fe-CDs on infected wound healing (Fig. 3C) [40]. Table 1 summarizes the applications of transition group metal-doped CDs in the field of antibacterials.

**Table 1:** Applications of transition group metal-doped CDs in the field of Antibacterials.

MCDs	Precursors	Application	λex/em	Ref.
Cu-CDs	acetic acid, EDA, CuCl_2_	Cu-CDs induce oxidative stress in the weakly acidic microenvironment of bacteria, leading to membrane disruption and ultimately bacterial death. Its excellent permeability and accelerated reactive oxygen species (ROS) generation give Cu-CDs excellent antibacterial potential, even at low doses.	340/465	[41]
Cu-CCDs	CuCl_2_·2H_2_O, ciprofloxacin	Compared with CCDs, Cu-CCDs showed superior antibacterial activity against *E.coli* and *S.aureus* with good bio-compatibility, suggesting that they have a promising future in clinical applications.	360/440	[42]
Cu/NCD	Melamine, citric acid, Cu(acac)_2_	Cu/NCD significantly inhibited the growth of E. coli cells under 365 nm light irradiation, while no significant inhibition was observed in the dark or when only copper-free CDs were used. Controlling the copper content in the CDs to a low loading of 1.09 wt Reduced the potential for leaching of toxic Cu^2+^.	–	[43]
AgNPs/CDs	Sliver nitrate, polyvinylpyrrolidone	Compared to AgNPs, AgNPs/CDs have higher redox properties with (MIC) of 40 μ g/mL for E. coli and 20 μ g/mL for S. aureus.	–	[44]
NSCDAg/NSCDAgAc	PEI, CA, AgNO_3_	The two CDs prepared exert antibacterial activity through membrane disruption and its effect on proteins. And they have multicolor imaging ability against Staphylococcus aureus.	360/452 350/466	[45]
Ag,N-CQDs	Citric acid, ascorbic acid, AgNO_3_, NH_3_∙H_2_O	Ultra-trace Ag atom doping reduces the cytotoxicity of Ag. Ag, N-CQD exhibits strong antibacterial properties against both *E.coli* and *S.aureus*, where it has a perforating effect on *S.aureus* bacterial membranes, whereas only strong chemisorption can work against *E.coli*.	330/390	[46]
Fe-CDs	Citric acid, FeCl_3_⋅H_2_O, urea	Fe-CDs interact with cell membranes to disrupt their integrity and enter bacteria, increasing intracellular iron levels, triggering an increase in reactive oxygen species (ROS) and leading to a glutathione (GSH)-dependent antioxidant damage mechanism. Excess ROS further lead to lipid peroxidation and DNA damage in the cell.	349/437	[47]
Fe-CDs	EDTA, FeCl_3_⋅6H_2_O	Fe-CDs with tunable fluorescence and enhanced peroxidase-like activity have led to the development of a novel "triple-play" multifunctional platform offering dual-mode/dual-target detection and near-infrared (NIR)-assisted antibacterial capabilities.	340/430	[48]
NiO NPs@C-dots	benzene-1,4-diamine, Ni(NO_3_)_2_ ⋅6H_2_O	The functional groups of NiO NPs@C-dots can increase the efficiency of antibacterial activity due to their interaction with receptor molecules (amino acids) and tend to damage bacterial cells.	–	[49]
Zn-CDs	Ethanol, *o*-phenylenediamine, L-tryptophan, zinc chloride	Zn-CDs maintained the catalytic oxidative properties of zinc metal and improved the bactericidal rate and ROS generation efficiency. The antibacterial regimen achieved up to 90 % bactericidal efficiency against S. aureus and has the potential to break through drug-resistant bacterial infections.	295/342 600/612	[50]
Zn-CDs	citric acid, ethylenediamine, zinc acetate	Zn-CDs produce reactive oxygen species (ROS) after 10 min of blue light excitation, which has the ability to inhibit bacterial growth and biofilm formation.	342/440	[51]
CQDs-ZnO	Zn(NO_3_)_2_·6H_2_O, graphite rods	CQDs-ZnO produces more than three times as many free radicals as pure ZnO under visible light irradiation, and has strong antibacterial activity, capable of killing more than 96 % of bacteria at a concentration of 0.1 mg/L.	–	[52]
QCuRCDs@BMoS2	Citric acid, CuCl_2_·2H_2_O	The long alkyl chains of QB and the sharp surface of MoS2 favour the destruction of bacterial structure, while electrostatic adsorption binds tightly to bacteria and shortens the bactericidal distance of reactive oxygen species (ROS).	370/442	[53]
Au@CD	HAuCl_4_, Chitosan, PVA	Au@CD exhibits excellent bio-compatibility and photothermal antibacterial activity. Under NIR irradiation, the inhibition rate was 99 + % against *S*. *aureus* and *E.coli*.	–	[54]
CDs@PtNPs(CPP)	PEI, PtNPs	CPP showed significant MRSA biofilm eradication and wound healing promotion in vivo via H_2_O_2_ in acidic infected tissues, while allowing sensitive and accurate detection of H_2_O_2_, antioxidants and pathogens.	540/655	[55]

Antibiotic resistance is an emerging global crisis, with microorganisms resistant to several clinically approved antibiotics becoming increasingly common. Therefore, efforts have been made to develop promising alternatives with broad-spectrum antibacterial activity and non-resistance. Although CDs are easily prepared, have acceptable biocompatibility, and are stable, their limited physicochemical and weak antibacterial properties limit further clinical applications of CDs. The physicochemical characteristics and antibacterial activity of the CDs are enhanced by metal doping, which exhibit tremendous antibacterial activity even in pathogens that are extremely difficult to remove. However, there are still some obstacles in the antimicrobial aspects, such as the inability to match with the corresponding bacterial types, the short action duration in vivo, and the resistance to bacteria that needs to be further investigated.

### 2.2 Cancer therapy

Cancer is one of the most widely spread diseases in the world, causing millions of deaths each year [56]. Traditional cancer therapies include surgery, radiotherapy and chemotherapy. Although the continuous development of cancer therapy in recent years has improved the overall survival rate of patients, there are still a number of obstacles. For example, surgical treatment faces individual differences and the difficulty of anatomical sites, which can lead to problems such as tumor metastasis and recurrence, and ultimately lead to the failure of cancer therapy [57]. Second, one of the primary causes of cancer therapy failure is thought to be the non-specific and indiscriminate destruction of normal tissues and cells other than cancer cells by radiation and chemotherapy. Another is the issue of multi-drug resistance brought on by prolonged chemotherapy. To overcome the problem of clinical drug resistance, many researchers have developed a variety of small molecule inhibitors and derivatives of natural compounds. For example, Manthena demonstrated that D-alpha-tocopherol polyethylene glycol succinate (TPGS) may inhibit the overexpression of P-glycoprotein (P-gp), thereby increase the bioavailability of BCS class II-IV drugs and improve drug therapeutic efficacy. However, most of them are limited due to reasons such as non-specificity and adverse side effects of the drugs [58,59]. CDs are very popular in tumor therapy due to their superior optical properties and easy functionalization of the surface. CDs are often used as a nano-diagnostic platform integrating optical imaging and drug therapy, which achieves optical imaging-guided drug-specific targeting and controlled release, improving the therapeutic effect of drugs while reducing the damage of drugs to other non-target organs [60]. Zhang et al. successfully synthesized OCDs from citric acid and (1R,2S)-2-amino-1,2-diphenylby-1-ol, which showed aggregation-induced emission properties and two-photon fluorescence imaging and generated a strong anticancer efficacy. This work demonstrates the importance of fluorescent CDs as smart materials for application in anticancer therapy [61]. Metal-doped CDs can regulate the charge density and form of charge transfer between CDs and metal ions, improve the physicochemical and optical properties of conventional CDs, and thus satisfy the accurate diagnosis of tumors, improve the efficiency of cancer therapy, reduce the incidence of drug resistance in cancer cells, circumvent the limitations of conventional therapies for cancer, and produce satisfactory effects in the field of cancer diagnosis and therapy.

**Fig. 4:**
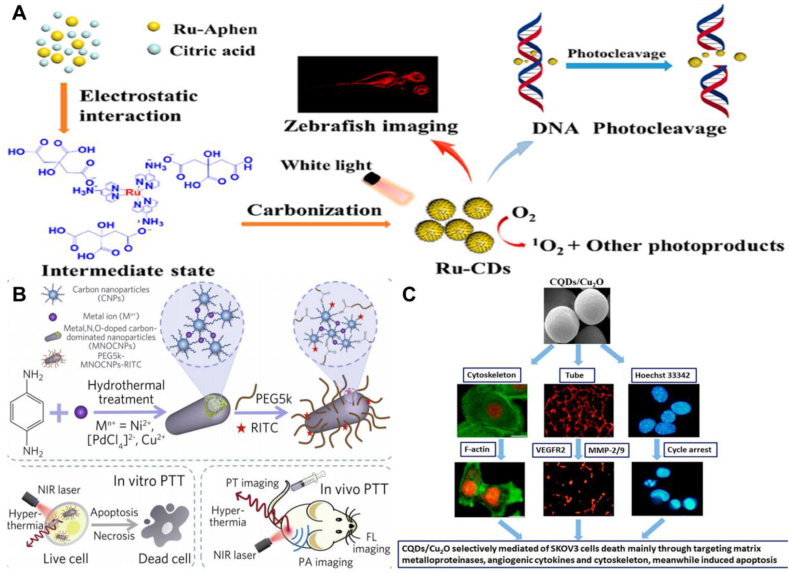
Cancer therapy application of transition group metal-doped CDs (A) Ru-CDs have promising biological applications for diagnostic and therapeutic purposes. Reprinted with permission from Yue et al. (B) MNOCNPs enable fluorescence (FL)/photobiological (PA)/thermal tri-modal imaging-guided PTT to monitor therapeutic response and maximize the efficacy of the nano-formulations. Reprinted with permission from Bao et al. (C) CQDs/Cu_2_O could be a potential therapeutic intervention for ovarian cancer SKOV3 cells. Reprinted with permission from Chen et al.

Yue et al. successfully prepared ruthenium-containing carbon dots (Ru-CDs) by a hydrothermal method using 5-amino-1,10-phenanthroline ruthenium (II) complex (Ru-Aphen) and citric acid as starting materials. Ru-CDs have been used not only as bio-imaging agents for the identification of tumor cells, but also as photodynamic nano-agents for cancer treatment due to their strong luminescence in water (QY = 20.79 %) and effective ROS generation. Photodynamic nano-agents for therapeutic purposes, this study successfully produced ruthenium-doped CDs with integrated diagnostic and therapeutic modes, which have promising biological applications (Fig. 4A) [62]. A novel trace carbon nanoparticles MNOCNPs were synthesized by a one-step hydrothermal method by Bao et al. A small amount of doping with metal ions endowed MNOCNPs with high extinction coefficients or photothermal conversion efficiencies. The nanoparticles have excellent photothermal properties and can be used for effective tumor ablation. It possesses high NIR absorption, enhanced cellular uptake, highly specific targeting ability and good biosafety. With their fluorescent probe coupling ability and inherent PA/PT properties, MNOCNPs enable fluorescence (FL)/photobiological (PA)/thermal tri-modal imaging-guided PTT to monitor therapeutic response and maximize the efficacy of the nano-formulation (Fig. 4B) [63]. Chen et al. successfully synthesized CQDs/Cu_2_O using CuSO_4_ and poly-vinylpyrrolidone, which for the first time achieved selective inhibition of ovarian cancer SKOV3 cells by targeting and regulating the cellular microenvironment. Compared to popular anticancer drugs, IC50 number of CQD/Cu_2_O is approximately 114-fold and 75-fold lower than the commercial artesunate (ART) and oxaliplatin (OXA) IC50s. The experimental results demonstrated that CQD/Cu_2_O selectively mediated ovarian cancer SKOV3 cell death mainly by decreasing the expression of MMP-2, MMP-9, F-actin, and VEGFR2, while CQD/Cu_2_O induced SKOV3 apoptosis through S-phase cell cycle arrest. All these findings reveal that CQD/Cu_2_O can be used as a potential therapeutic intervention for ovarian cancer SKOV3 cells (Fig. 4C) [64]. Table 2 summarizes the applications of transition group metal-doped CDs in the field of cancer therapy.

**Table 2:** Applications of transition group metal-doped CDs in the field of Cancer therapy.

MCDs	Precursors	Application	λex/em	Ref.
*Cu, N-CDs*	EDTA·2Na, CuCl_2_	Cu^2+^ imparts NIR absorption to generate heat and reactive oxygen species with a strong luminescence peak at 436 nm under 376 nm excitation, which can be used as an infrared thermography agent and fluorescence cell imaging agent.	376/436	[65]
CCD NPs	Cu(NO_3_)_2_, citric acid, thiourea, DOX	The assembled CCD NPs were responsive to GSH and acidic pH in the tumor microenvironment, resulting in the specific release of DOX, Cu^2^⁺and CDs. The CCD NPs had excellent bio-compatibility and synergistic anti-tumor capabilities, and completely inhibited the growth of 4T1 tumors.	–	[66]
Co-CDs	vitamin B12, citric acid	Co-CDs show peroxidase-like activity and can specifically catalyze the generation of multiple reactive oxygen species from H_2_O_2_ in cancer cells, leading to cancer cell death.	340/460	[67]
Fe@EDTA-CDs	EDTA, o-PD, Fe^3+^	The chromophores in the carbon nuclei showed strong red fluorescence under visible light excitation, which enhanced the catalytic activity of the surface Fe ligand sites, and had a high ROS generation rate and photomodulated anticancer efficiency.	570/642	[68]
CDs@EDTA@Gd@Fe	*p*-phenylenediamine	Based on the Fenton reaction, CDT shows excellent anticancer effects in vitro and in vivo by releasing Fe^2+^ in tumors. It also exhibits bright and stable fluorescence and strong T1-weighted MR imaging (MRI) contrast.	550/620	[69]
PMQDs	VCQDs	PMQDs can be used as highly efficient photosensitiser, ROS are efficiently generated under light, can hinder the Nrf2 antioxidant program to enhance cancer biodynamic therapy.	360/445	[70]
C-CD/TiO_2_	BA, PEG, DMA, TiO_2_	Modulating the energy band gap through the photocatalytic activity of TiO_2_, C-CD/TiO_2_ showed up-regulation of tumor pro-apoptotic markers such as P53 and BAX, with excellent direct anti-cancer activity.	–	[71]
MWCNT/CQD/MnO_2_	–	MWCNT/CQD/MnO_2_-1 and MWCNT/CWD/MnO_2_-2 exhibited good cytostatic properties against Hep2 cell lines and can be used as good photocatalytic as well as anticancer agents.	–	[72]
RuCN/(N-)CDs	RuCN	Ru balance the anticancer efficiency and resulted in better anticancer outcomes by reducing the oxidative stress associated with cancer progression.	430/560	[73]
CDs@RuCNN-CDs@RuCN	–	N-CDs@RuCN interacted most actively with the Wnt signalling pathway in A2780 and with PI3–K/Akt in CAL72 cells.	–	[74]
Au/GdC	Gold (III) trihydrate, GdCl_3_, N-acetyl-l-cysteine	Au/GdC nanocomposites exhibit paramagnetic properties, surface plasmon resonance in the NIR, high longitudinal relaxation rate (r1 = 13.95 mM^−1^ s^−1^), and excellent photostability, which can effectively destroy cancer cells.	–	[75]
V−CDs	NH_4_VO_3_, Citric acid	Once V-CDs enter the tumor, their fluorescence imaging ability is stimulated by the reaction between vanadium and overexpressed H_2_O_2_ in the tumor microenvironment. Meanwhile, hydroxyl radicals generated by the catalytic reaction of V-CDs induce oxidative damage in tumor cells with less cytotoxicity and side effects on normal cells.	-/560	[76]

Cancer is one of the most common diseases in the world, and the failure to detect it early and the inability to target treatment are the main reasons for the low survival rate of cancer patients. Currently, medical imaging technologies for cancer diagnosis still face limitations such as poor biocompatibility and inability to bypass biological barriers. Traditional chemotherapeutic drugs also face challenges such as toxicity issues and inability to accurately target therapy. In recent years, CDs are often used as a nano-diagnostic platform for optical imaging and drug therapy. Metal doping improves the optical properties and cancer therapeutic effects of CDs to achieve precision targeted therapy guided by optical imaging, improve cancer therapeutic effects, and reduce damage to non-target tissues and organs. However, the organism's immune response and the toxicity issue are the most important factors to take into account before clinical translation.

### 2.3 Osteogenesis

According to the World Health Organization, trauma is a global public health problem. Approximately 3–9 million people are injured each year in developed countries. With the development of the economy and transport, nearly 1.2 million people die as a result of trauma caused by traffic accidents. In the last 30 years, the number of deaths due to trauma has doubled in European countries [77]. Open comminuted fractures can result from severe trauma, which is usually associated with severe soft tissue injury, bone defects, infection and eventually osteonecrosis. In addition to trauma, large or minor bone defects can also be caused by inflammation, cancer, or other space-occupying lesions. Bone has a remarkable endogenous regenerative repair capacity, even in advanced age, metabolic slowdown, immunodeficiency or other degenerative diseases, etc., can be completely regenerated without the formation of scar tissue and can be restored to a better form and function [78,79]. Sufficient volume of new bone tissue is essential for both musculoskeletal performance and bone shape restoration. The repair of bone defects depends mainly on the size of the bone defect, when the size of the bone defect area is greater than or at a critical value, the fibrous connective tissue, which migrates faster than osteoblasts, occupies the main component of the bone defect repair, which ultimately leads to the failure of self-repairing [[80], [81], [82]]. When bone self-repair fails, bone grafting—which can involve autologous bone grafting, allogeneic bone grafting, and xenogeneic bone grafting—becomes the second alternative. Autologous bone grafting is considered the "gold standard" of bone grafting [83]. However, autografts have a number of limitations. Such as pain and secondary infections in the donor area [84], Insufficient available bone volume [85] and survival and resorption of grafted bone. Therefore, finding a new biomaterial with an osteogenic function is crucial for the therapeutic repair of bone defects. In recent years, as a matrix or main component, CDs have shown promising applications in the repair of bone defects. Jin et al. generated ascorbic acid carbon dots by a one-step microwave-assisted method, and their experimental results showed that this CDs induced an increase in intracellular calcium to activate the ER stress and PERK-eIF2α-ATF4 pathways, which effectively promotes in vitro osteogenic differentiation, enhances matrix mineralization in vivo in a model of cranial defects, and facilitates regeneration of new bone, which has the potential to be used for repairing bone defects in clinical practice [86]. At the same time, the restorative effect can be further improved by changing the CDs or mixing them with other osteogenic enhancers or materials [87]. Liu et al. synthesized a novel N-acetyl-l-cysteine (NAC)-derived red fluorescent carbonylated polymer dots (CPDs), NAC-CPDs were able to target accumulation in the alveolar bone in vivo and reduce the level of alveolar bone resorption in mice with periodontitis, and modulate redox homeostasis and promote bone formation in the periodontitis microenvironment through modulation of the Kelch-like ECH-associated protein l (Keap1)/nuclear factor red factor 2-associated factor 2 (Nrf2) pathway [88]. However, the osteogenic capacity of CDs alone is limited. Many metals, such as Cu, Mg, and so on, have their unique osteogenic potential. Taking advantage of the easy functionalization of CDs, metal doping improves the physicochemical properties and osteogenic capacity of CDs. At the same time, the synergistic bone regeneration effect of CDs and metals provides a new therapeutic solution to address the clinical issues associated with bone defect repair.

**Fig. 5:**
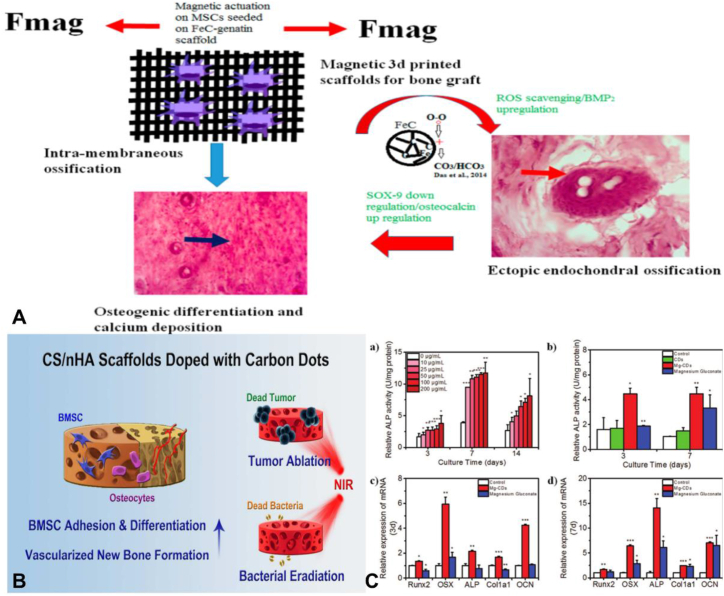
Osteogenesis application of transition group metal-doped CDs (A) Proposed mechanism for endochondral ossification in FeCD−gelatin composite scaffold Reprinted with permission from Das et al. (B) CS/nHA/CD enhances osteoinductive properties of bone repair scaffolds, and have potential applications. Reprinted with permission from Lu et al. (C) Effects of Mg-CD on ALP activity and mRNA related gene expression. Reprinted with permission from Yang et al.

Das et al. synthesized CD-SPION composite nanoparticles (FeCD) using a simple microwave-assisted technique. PCR studies after co-culture with MSC showed that MSCs expressed gene expression of both bone and cartilage specific markers, suggesting that FeCD driven by magnetic field could enable MSCs to enter the endochondral osteogenic pathway through differentiation. After the co-cultured MSCs were implanted subcutaneously, the obtained tissues showed the characteristics of bone formation and cartilage differentiation in vivo. In conclusion, the doping of superparamagnetic iron oxide nanoparticles in CDs not only enhances the field of bioimaging through complementary imaging modalities such as fluorescence imaging and magnetic resonance imaging, but also provides a new way for the study of osteochondral differentiation through its application in three-dimensional nanocomposite scaffolds(Fig. 5A) [89]. Lu et al. prepared a novel cadmium-doped chitosan/nano hydroxyapatite (CS/nHA/CD) scaffold by freeze-drying. Compared with pure CS/nHA scaffolds in vivo, CS/nHA/CD upregulated genes involved in focal adhesion and osteogenesis, enhanced cell adhesion and osteoinductivity of rat bone marrow mesenchymal stem cells, and significantly improved vascularized neoplastic bone tissue formation at 4 weeks. Meanwhile, the expression of focal adhesion kinase (FAK) and vinculin (VCL) was significantly higher in CS/nHA/CD scaffolds than in CS/nHA scaffolds (p < 0.01 and 0.05) The scaffolds were also applied for photothermal treatment of cancer under near-infrared irradiation and had significant antibacterial properties. These results suggest that cadmium-doped can enhance the osteoinductive properties of bone repair scaffolds and that CD-doped scaffolds have potential applications in PTT(Fig. 5B) [90]. Yang et al. prepared a new Mg^2+^ doped CDs (Mg-CDs) by a one-step hydrothermal method using metal gluconate as raw material for the first time. The prepared Mg-CDs can promote the differentiation of osteoblasts by increasing the activity of ALP and up-regulating the expression of related mRNA. Mg-CDs possess the ability to label cells due to their multicolor fluorescence properties. This study introduces a new candidate material for osteogenic biomaterials by introducing a bifunctional bioessential metal ion doped CDs, and provides valuable insights into the potential applications of Mg-CDs in the field of cellular labeling and osteogenic biomaterial development (Fig. 5C). [91]. Table 3 summarizes the applications of transition group metal-doped CDs in the field of osteogenesis.

**Table 3:** Applications of transition group metal-doped CDs in the field of Osteogenesis.

MCDs	Precursors	Application	λex/em	Ref.
Ca/P-CDs	ethanolamine phosphate, alcium gluconate	Ca/P-CDs have stable excitation-dependent emission spectra and well dispersion in water, upregulated the expression of bone differentiation-related genes, including ALP, RUNX2 and OCN.	396/480	[92]
Mg-CDs	Lycium ruthenicum, MgCl_2_	The appropriate concentration of Mg-CDs is suitable for long-term cell growth, and the cells have better growth, proliferation and osteogenic differentiation ability, showing great potential in bone tissue regeneration.	465/537	[93]
PCL/PVA-TCP-CD	ammonium hydrogen citrate, Gallus egg-shells	The addition of only 1 w t% of CDs and TCP3 to PCL/PVA increased ALP activity and cell proliferation rates, while the synergistic effect of CDs/TCP3 resulted in the highest rates of osteogenic differentiation and proliferation compared to other scaffolds.	400/463	[94]
Zn-CDs	zinc gluconate	the results of in vivo experiments showed that the volume of in vivo bone defect repair in the Zn-CD/GH group was twice as much as that in the control group.	374/466	[95]
ZnO-BGN	ZnO QDs	ZnO-BGN improved the osteogenic differentiation of hMSC with its apatite-forming ability, unique ion-releasing behavior, potent antibacterial activity, non-cytotoxicity and osteogenic potential as a potential material for bone regeneration.	–	[96]
Zn-CD	zinc gluconate	Compared with the raw material Zn-G, Zn-CDs induced osteoblast differentiation effects in terms of significantly increased gene expression and ALP activity in MC3T3-E1 cells. In addition, Zn-CDs can be applied as bifunctional nanomaterials with multicolor bioimaging capabilities.	360/450	[97]
f-CDs-PE-Au@HA	f-CDs, polyethylene, Au, apatite	The use of f-CDs-PE-Au@HA coating BC2 improved the binding and proliferation of cells on the biocomposite coat, showing strong alkaline phosphatase activity.	–	[98]

Bone regeneration involves all stages of human growth and development, and the interaction between osteoblasts, which produce new bone, and osteoclasts, which resorb old bone, maintains a constant dynamic balance. The repair of bone defects is a major clinical challenge, and traditional treatment of bone defects is limited by the insufficient supply of autologous bone, immune rejection, and high medical costs. Small size and biocompatible CDs have received much attention from researchers. The synergistic effect of metal doping enhances the osteogenic ability of CDs. However, the osteogenic mechanism of metal-doped CDs still requires further in vivo studies, and the optimal dose to promote osteoblast differentiation is not clear, and the CDs are rapidly cleared from the body when injected intravenously or directly, all of which need to be seriously considered.

### 2.4 Biosensing

CDs are relatively new fluorescent nanomaterials that have outstanding applications in several different fields due to their excellent bio-compatibility, controlled photoluminescence (PL), high quantum yield (QY), and unique electronic and physicochemical properties that outperform other contributors to the carbon isomorphisms, so they have outstanding applications in many different fields [99]. The photoluminescence mechanism of CDs has been extensively studied, mainly attributed to the surface, carbon-nucleated, and molecular states as well as their synergistic effects. Due to their photoluminescent properties, CDs can be used as electron donors and acceptors [100]. Due to their upconversion photoluminescence (UCPL) characteristics, CDs are capable of emitting high-energy short-wave photons through the absorption of multiple low-energy long-wavelength photons [101]. Interactions between CDs and chemical substances often lead to Fluorescence quenching or enhancement of CDs, and a large number of studies have demonstrated the irreplaceable role of CDs as sensors for the detection of various substances or environments [102]. For example, as an effective fluorescent sensing platform for metal ion detection [103,104], as a cellular microenvironmental pH sensing probe [105,106], as tetracycline or toxic pollutants for Versatile fluorescent probe for antibiotics [107,108]. Recently, the improvement of physicochemical properties of CDs by CDs doping and surface passivation has attracted much attention from researchers. CDs are further developed in the field of biosensing by metal doping and controlling their size, morphology, structure and bandgap energy to enhance their physicochemical properties, visible light absorption probability and quantum yield, etc.

**Fig. 6:**
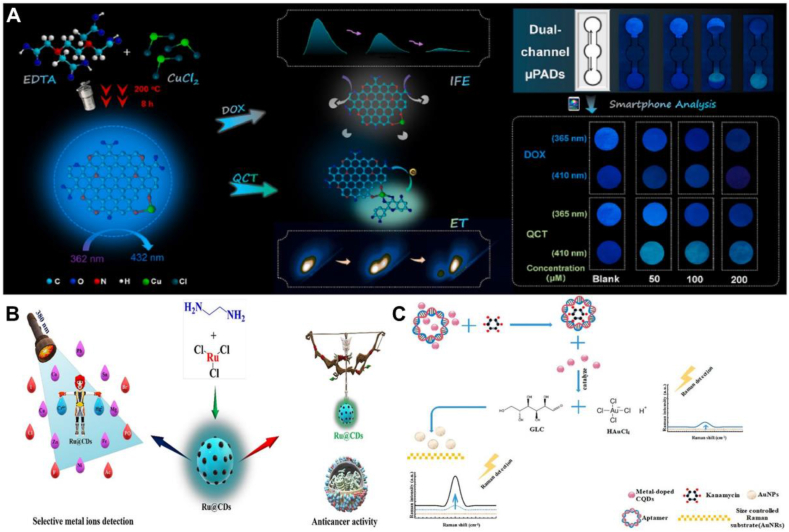
Biosensing applications of transition group metal-doped CDs. (A) N, Cu-CDs provide a new pathway for effective detection of TCs and QCTs. Reprinted with permission from Li et al. (B) Ru@CDs provide a new technique for exploring a versatile fluorescent probe for a better environmental pollutant detector and bioimaging agent. Reprinted with permission from Arun et al. (C) Zn-doped CQDs exhibit high sensitivity, good specificity and wide linear detection range for the detection and quantification of kanamycin. Reprinted with permission from Wang et al.

Due to the emergence of multidrug-resistant *E.coli*, tetracyclines (TCs) and quercetin (QCT) have become more common therapeutic modalities used to combat recalcitrant bacterial infections; however, the long-term accumulation of TCs and QCTs poses a potentially harmful effect on human health and the ecosystem. Therefore, the effective detection of TCs and QCTs is a matter of urgent concern. Li et al. prepared a novel fluorescent probe based on nitrogen and copper co-doped carbon dots (N, Cu-CDs) by hydrothermal method using ethylenediaminetetraacetic acid (EDTA) and anhydrous copper chloride as precursors. Due to the internal filtering effect (IFE), the fluorescence intensity of N, Cu-CDs was directly quenched and the detection limits obtained by single-signal fluorescence sensing were as low as 8.37 nM for DOX, 2.43 nM for TC, 8.28 nM for OTC and 8.410 nM for CTC. Based on the electron-transformation (ET) process occurs, another emission at (490 nm, 10 nm) can be used as a three-dimensional proportional fluorescent probe for the detection of QCT. In addition, a dual-channel fluorescence sensing platform based on a microfluidic paper-based analysis device (μPAD) was developed for simultaneous visual discrimination of TC and QCT. This study provides a new approach for the effective detection of TCs and QCT(Fig. 6A) [109]. Arun et al. used a one-step microwave method using ruthenium chloride and ethylenediamine to prepare Ru@CDs with a quantum yield of 19.21 %, which has an extremely low lower limit of detection for Cr^6+^ and Hg^2+^ in aqueous environments compared to CDs, as low as 0.128 mM and 0.328 mM, respectively. Meanwhile, Ru@CDs showed good cytotoxicity, effective growth inhibition of cancer cells, and selective use as effective bioimaging agents. This work readily provides a new technique for exploring a versatile fluorescent probe as a better detector of environmental pollutants and bioimaging agent (Fig. 6B) [110]. Wang et al. developed a novel SERS sensor for the analytical detection of kanamycin, in which Zn-doped CQDs were prepared with catalytic properties and were used to enhance the aptamer-controlled on/off switching of the SERS signal. In the absence of a target, its catalytic activity was inhibited by the nucleic acid aptamer wrapped around the surface. Upon binding of the aptamer to kanamycin, the Zn-doped CQD was released, resulting in catalytic activity on the surface of the Au nanorods to reduce Au3+ to Au, thus enhancing the SERS signal of kanamycin. Under the optimized detection conditions, the sensor showed high sensitivity, good specificity and a wide linear detection range of 10^−12^ to 10^−5^ g mL^−1^ for the detection and quantification of kanamycin, with a detection limit of 3.03 × 10^−13^ g mL^−1^. Simultaneous detection with a kanamycin-like antibiotic and an interfering agent demonstrated the excellent selectivity of the sensor (Fig. 6C) [111]. Table 4 summarizes the applications of transition group metal-doped CDs in the field of Biosensing.

**Table 4:** Applications of transition group metal-doped CDs in the field of Biosensing.

MCDs	Precursors	Application	λex/em	Ref.
Ru: CNDEDAs	CA, Rucl_3_	PL quenching of Ru: CNDEDAs upon addition of model quinones (MQ) demonstrated their utility as toxic quinone sensors in HeLa cells.	–	[112]
Ag Np-CQDs	citric acid, ammonium sulfate, AgNO_3_	The efficiency of Ag nanoparticles-CQDs for gemcitabine assay was much better than other methods, and the gemcitabine quenched the Ag Np-CQDs fluorescence by light-induced charge transfer.	320/430	[113]
N-CQD@Fe_2_O_3_/MWCNT/GCE	FeCl_3_·6H_2_O, EDA, CA	The nanocomposites exhibited low charge transfer resistance and rapid electron transport on their surfaces. The detection of UA, XA and 5-FU showed excellent sensitivity with detection limits of 0.106, 0.092 and 0.019 μ M, respectively.	–	[114]
Cu, N@CQDs	CuSO_4_·5H_2_O	For the colorimetric method, the absorption intensity increased linearly over the concentration range of 4.3–110.0 μ M, with a LOD of 1.3 μ M. For the fluorescence method, the emission intensity of Cu N@CQDs decreased linearly with the addition of AMX over the concentration range of 0.2–120.0 μ M, with a limit of detection LOD of 0.06 μ M.	370/440	[115]
Cu-CDs	Citric acid, urea, copper acetate	Cu-CDs showed effective peroxidase-like catalytic activity at pH 7 for TMB and pH 6 for ABTS. A colorimetric sensing platform for the detection of ascorbic acid was produced based on the reversible reduction of oxidized TMB.	–	[116]
D-CDs	1-(2-pyridylazo)-2-naphthol, Copper (II) Chloride Dihydrate	When λex is 260 nm–320 nm, the emission peak of D-CDs is located around 370 nm. However, at the excitation wavelengths of 340–360 nm, the emission peaks are red-shifted to the visible region with two peaks (426 nm/488 nm), which has good potential for applications in dual-signal analysis and visual perception.	350/426 and 488	[117]
Cu-CDs	CA, Cu(NO_3_)_2_·3H_2_O	Cu-CD Exhibits excellent peroxidase-like activity and remains stable over a wide pH and temperature range, Glucose can be detected selectively and sensitively using Cu-CD chemiluminescent sensing.	–	[118]
GT/CS/Cu based CDs	Chitosan, copper (II) sulfate, FeCl_3_·6H_2_O	Fe^3+^ combines with CDs and the fluorescence intensity of CDs is quenched due to the electron transfer process. Later, by introducing isoniazid into the CDs-Fe^3+^ system, Fe^3+^ is converted to Fe^2+^ and fluorescence is restored. The new method has a high capability to detect low concentrations of isoniazid even in the presence of other drugs and interfering substances.	360/465	[119]
N, Zn-CDs	Citric acid, Zn(O_2_CCH_3_)_2_(H_2_O)_2_	Based on the Fenton reaction mechanism for the detection of hydrogen peroxide, it displays sensing activity over a wide concentration range associated with a linear range of 10–70 μ M, with detection limits as low as 8 n M.	380/510	[120]
Zn-CDs	Sodium citrate, Zinc chloride, ferrous sulfate heptahydrate	Zn-CDs showed high sensitivity and response to hydrogen peroxide and glucose over a wide concentration range, with linear ranges of 10–80 μ M and 5–100 μ M, respectively.	350/440	[121]
Zn-CDs	sodium citrate, Zinc chloride	Zn^2+^ acts as a surface passivator to prevent graphene π-π stacking aggregation, leading to an increase in the QY of Zn-CDs. Zn-CDs was used for the ultra-trace detection of Hg^2+^ with a detection limit of 0.1 μ M.	340/440	[122]
Zn-CDs	Zinc chloride, *p*-Phenylenediamine	By controlling the ratio of precursors, the luminescence characteristics were changed and the emission wavelength was adjusted between 610 nm and 390 nm, and multicoloured zinc atom-doped CDs were successfully synthesized for the detection of water and organic solvents.	–	[123]
CDs-Zn Cd Te	cadmium acetate, zinc acetate,	The detection limit of guanine was 0.076 μ mol⋅L^−1^ (3σ/k), and the linear range was from 0.25 to 40.0 μ Mol⋅L^−1^.The smart ratiometric fluorescent probe was demonstrated to be useful for the detection of guanine in DNA samples.	–	[124]
MgCQD, ZnCQD	Hen feather MgSO_4_ Zn (NO_3_)_2_·6 (H_2_O)	At pH 7 in the presence of 20 ppm Hg, MgCQD and ZnCQD exhibited 70 % and 52 % fluorescence quenching, respectively. At pH 2 in the presence of 20 ppm 4-NP, the fluorescence quenching of MgCQD and ZnCQD exceeded 4 %.	320/413, 316/390	[125]
CDs/MgO/SPCE	Magnesium (II) nitrate, Red Korean ginseng	CDs/MgO nanocomposites showed excellent electrochemical oxidation efficiency compared to bare CDs. Among them, CDs-5.0/MgO nanocomposites showed the maximum oxidation response to DOX (1 μ M) detection at pH 10 and the scan rate of 5 mV/s.	–	[126]
Mn, N-CQDs	branched polyethyleneimine, manganese chloride,	Folic acid has a sensitive and selective fluorescence quenching with a low detection limit and co-existing molecules that do not belong to the folate family do not cause any detectable changes.	360/442	[127]
Mn-CDs	Sodium citrate, Citric acid, Manganese (II)carbonate	An extremely sensitive method for the detection of heavy metal Mercury was developed by adding Hg to study the reversible switchable fluorescence property Hg^2+^/S^2−^ of Mn-CDs.	340/440	[128]
Pd-CQD/Pt-CQD	Pd[II]TPP or Pt[II]TPP, nitric acid, EDA	The QY of CQD, Pd-CQD and Pt-CQD were 1.17 %, 8.15 % and 15.2 %, respectively. In addition, they can be used as fluorescent probes for the specific and sensitive detection of Fe^3+^ in aqueous solutions.	360/460	[129]
CD/CeO_2_	ammonium cerium nitrate, urea, taurine	Compared to bare CDs, CD/CeO_2_ facilitated the electron transfer at increased CDs concentrations, and the CDs-5.0/CeO_2_/SPCE sensing system with 5 w t% CDs showed the highest oxidative response to DOX (5 μ M) at pH 5, and excellent selectivity for DOX in the presence of common interferents.	340/430	[130]

CDs are relatively new fluorescent nanomaterials, and their interactions with chemical substances often lead to fluorescence burst or enhancement of CDs. A large number of studies have shown that CDs have irreplaceable roles as sensors for detecting various substances or environments. Novel metal-doped CDs-based sensors enhance the intrinsic properties of CDs with good stability, selectivity and broad detection limits for a wide range of contaminants. However, Precise control and additional process optimization are hampered, because the complete mechanism and fundamentals of the doping process are not yet fully understood. In addition, the recycling of metal-doped CDs after use is a major concern.

### 2.5 Bioimaging

CDs have a number of significant features, including low toxicity, considerable bio-compatibility and permeability, weak interactions with proteins, resistance to solubilization and photobleaching, ease of removal from the body, low cost, and immune system evasion, which are a solid basis for the role of CDs in diagnostics and imaging [131]. Due to their small particle size, photostability, bio-compatibility and non-flickering fluorescence and adjustable color, CDs have great potential for fluorescence bioimaging and multimodal bioimaging of cells and tissues, and in recent years, biomedical imaging has become one of the most promising and frequently used areas for CDs [[132], [133], [134], [135]]. Jiang et al. used neutral red and levofloxacin as precursors to synthesize a novel RNA-targeted red-launching carbon dot (M-CD), which could be rapidly internalized into cells within 5s and imaged in real time to visualize the dynamic processes of intracellular stress granules under oxidative stress. M-CDs pave a new way to visualize RNA dynamics and study phase separation behavior in living cells [136]. Modification is one of the most critical technologies to improve the application of biomedical materials; heteroatom doping, surface modification, and size control improve the photoluminescence properties of CDs by increasing the number of energy traps that lead to surface defects. Currently, various functional ligands are currently used for surface modification of CDs, including polymers, organic molecules, ions, DNA and proteins [137]. Among them, metal ions, due to the presence of valence electrons and their electron transfer process, the holes on the surface of CDs are radiolytically complexed with electrons, which improves the QY and enhances the effect of photoluminescence of CDs, opening a new path in biomedical imaging fields such as drug delivery, in vivo tracing, and the development and utilization of contrast agents [138,139].

**Fig. 7:**
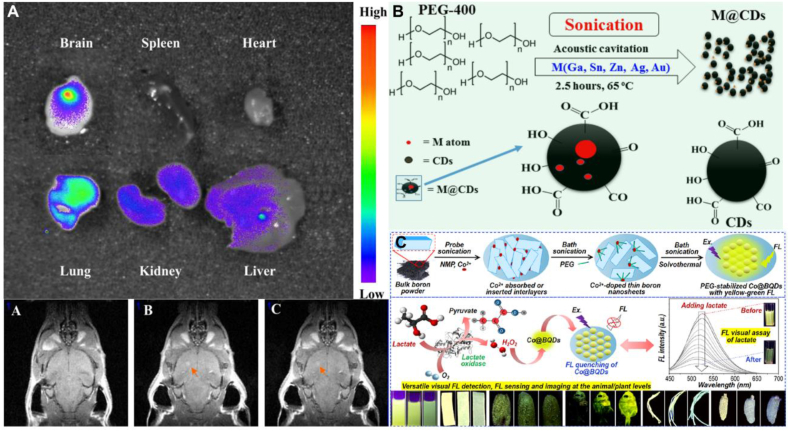
Bioimaging applications of transition group metal-doped CDs (A)Mn-doped CDs enhance MR/optical imaging contrast in glioma regions and offer great promise for the detection and intraoperative localization of microscopic brain gliomas. Reprinted with permission from Ji et al. (B) M-CDs are best suited for cellular labelling and have no negative effect on neuronal differentiation and growth. Reprinted with permission from Kumar et al. (C)Co@BQDs show long-term tracking and bioimaging capabilities at the live cell and small animal level. Reprinted with permission from Yang et al.

Gliomas are life-threatening diseases with low survival rates. Early detection and accurate intraoperative localization of gliomas is crucial for improving prognosis. Using citric acid, urea and manganese chloride as raw materials, Ji et al. synthesized Mn-doped CDs with sizes less than 5 nm, which showed different excitation-dependent PL emission and low cytotoxicity, Mn^2+^ provide CDs with effective r1 relaxivity for MR imaging. In vivo MR imaging and in vitro optical imaging of mouse microgliomas showed that Mn-doped CDs enhanced the MR/optical contrast in the glioma region, providing the possibility of detection and intraoperative localization of microgliomas, revealing the great promise of Mn-doped CDs as a MR/optical dual-modal imaging nanoprobe for the detection and intraoperative localization of microgliomas(Fig. 7A) [140]. Kumar successfully prepared CDs doped with five different metals (Sn@CDs, Ag@CDs, Zn@CDs and Au@CDs) by an acoustic chemical process. The experiments demonstrated that M-CDs have high QY, high dispersibility, stable size uniformity and low cytotoxicity, were shown to interact efficiently with neuron-like cells, were optimal for cell labelling, had no negative impact on neuronal differentiation and growth, and provided new insights into the production of inexpensive, highly sensitive and selective materials for a variety of biomedical applications (Fig. 7B) [141]. Yang et al. prepared Co@BQDs and BQDs using boron powder, N-methylpyrrolidone and aqueous cobalt nitrate solution. Based on the formation of B–Co bonds and the inhibition of B–O reactions, Co@BQDs showed higher colloidal and FL stability than BQDs. This work performed FL imaging measurements using visual FL colors by immersing fresh willow shoots, enoki mushrooms and live glassfish in Co@BQDs. Based on enzymatic and cascade oxidation of Co@BQDs, the nanoprobes exhibits a selective and sensitive FL response to lactate and is capable of effective FL sensing of lactate in biological samples by FL spectroscopic measurements. The probe enables versatile FL imaging and visual FL semi-quantitative detection of lactic acid in both liquid-phase (colorimetric cups) and solid-phase (filter paper strips, latent fingerprints) systems. Thus Co@BQDs can be used as new efficient imaging agents and show great promise for long term tracking and bioimaging at the live cell and animal level (Fig. 7C) [142]. Table 5 summarizes the applications of transition group metal-doped CDs in the field of bioimaging.

**Table 5:** Applications of transition group metal-doped CDs in the field of Biosensing.

MCDs	Precursors	Application	λex/em	Ref.
Mn-CDs	O-phenylenediamine, manganese acetate tetrahydrate	Mn-CDs have stronger fluorescence properties than Mn-free CDs and exhibit high T1-weighted MR imaging performance with a longitudinal relaxation rate of 12.69 mM^−1^-s^−1^. They can be used as bimodal nanoprobes for fluorescence and MR imaging.	420/580	[143]
Mn-CDs	Manganese (II)phthalocyanine	The Mn-CDs assembly can be used as near-infrared fluorescence (FL) (maximum peak at 745 nm) and T1-weighted magnetic resonance (MR) (relaxivity value of 6.97 mM^−1^s^−1^) imaging. Simultaneously, the oxygen concentration was increased under acidic H_2_O_2_ conditions to achieve bimodal FL/MR imaging and enhanced PDT.	690/745	[144]
CDs/MnO_2_-PEG nanohybrids	potassium permanganate	MRI capability is not available in normal physiological environments. However, in the tumor microenvironment, strong MRI signals can be detected due to the highly sensitive response of manganese dioxide to acid/hydrogen peroxide, the recovery of CDs fluorescence, and the simultaneous generation of single-line oxygen.	–	[145]
Mn-CQDs@FA/Ce6	Waste green tea powder, MnCl_2_	The composite specifically targets human epithelial cancer cell line (HeLa) cancer cells overexpressing the folate receptor, while the enhanced MRI signal has an r2/r1 ratio of 5.77. It can be used as a dual-modality FL/MRI probe and a highly effective PDT agent for both remote detection and eradication of cancer cells.	–	[146]
MnZnS/CuZnS	PDCA, Alcl_3_, Zn^2+^, Cu^2+^, Mn^2+^	The quantum dots were delivered in the nucleus and cytoplasm of human breast cancer cells (MCF7) for bioimaging and biomarkers.	–	[147]
Mg-CDs	Tamarinds, l-Arginine, Mg (CH_3_COO)_2_	Stable and tunable color in aqueous solution, very high luminescence quantum yield (54.16 %), easy penetration into cells for live cell imaging.	360/-	[148]
C-dot/Ag composite nanoparticles	l-arginine, silver nitrate	The presence of Ag nanoparticles and the higher nitrogen content resulted in a redshift of the excitation and emission intervals compared to CDs. C-dot/Ag composite nanoparticles showed superior bactericidal ability than CDs and were successfully used for fluorescence imaging of Escherichia coli.	370/455	[149]
Cu:CdS@ZnS	–	Cu-doped CdS quantum dots have higher fluorescence brightness and photostability, and concentration burst or aggregation burst is largely relieved, leading to improved cellular imaging.	425/630	[150]
Cu:CdS QDs	CdCl_2_· 2.5H_2_O, CuCl_2_· 2H_2_O, l-cysteine,	With ultra-small size (∼5 nm), high QY (25.6 %), near-infrared emission (∼700 nm), and low cytotoxicity, it was successfully applied as a fluorescent probe for labelling live 3T3 cells.	–	[151]
64Cu-doped CdSe/ZnS QDs	CuCl_2_	High tumor targeting ability was shown in U87MG glioblastoma xenografts, and luminescence imaging of tumors in the absence of excitation light has feasibility in vivo.	–	[152]
PEG-RLS/Fe@CDs	DMF, iron (II) phthalocyanine,	As a quad-modal contrast agent for fluorescence/photo-acoustic/photothermal/magnetic resonance imaging, Fe@CDs improves gene therapy through transfection enhancement, real-time tracing and tumor synergistic disruption.	–	[153]

Bioimaging plays an important role in diagnosis and treatment in the biomedical field. Compared to conventional inorganic and organic fluorophores, CDs are considered superior candidates for bioimaging due to their coordinated emission, good biocompatibility and excellent resistance to photobleaching. Metal doping enhances the optical properties of CDs, such as pronounced excitation-dependent PL emission, improved photoluminescence quantum yield, and increased longitudinal chirality, offering possibilities for disease diagnosis and intraoperative localization. However, the selection of suitable in vivo models for repeated validation is necessary before further translation to clinical applications.

### 2.6 Conclusion

As an emerging class of fluorescent nanomaterials, CDs have received great attention from researchers due to their superior nanoscale effects, excellent photoluminescence properties, strong water solubility and good biocompatibility. transition group metals are cofactors for many life-critical proteins and are essential for all life forms, yet their biosafety and utilization remain a major concern. By doping transition group metals, not only the physicochemical properties of CDs can be enhanced to a certain extent, but also the challenges of biosafety and utilization of transition group metals can be greatly improved, which has a broad application prospect in the biomedical field. However, there are still certain problems with metal-doped CDs. For example, more research is needed to understand the antibacterial mechanism against multidrug-resistant bacteria; the integrated platform for cancer diagnosis and treatment needs to be improved; biosensing and bioimaging accuracy needs to be increased; and more attention needs to be paid to the toxicity of metal-doped CDs.

## 3 Biomedical applications of rare-earth group metal-doped CDs

Rare-earth group is the abbreviation of a group of metals, rare-earth elements have been discovered since the end of the 18th century, a total of 17 elements, including 15 lanthanides and two elements closely related to the lanthanides, scandium (Sc) and yttrium (Y), have a wide range of unique applications in high-tech, biomaterials, communications and industries [154]. Rare-earth group metals have unique spectral properties, including optical stability, long fluorescence lifetimes, large Stokes shifts, and sharp emission bands, which have attracted much attention in recent years [[155], [156], [157]]. Addition of rare-earth group metals (Gd^3+^, Eu^3+^, Hf^3+^, Ho^3+^, etc.) to CDs is an effective strategy to improve the physicochemical properties of CDs [158]. Transition group metal-doped CDs have been a major research boom in the field of biomedical materials in recent years, while rare-earth group metal-doped CDs have been relatively little studied. As a result, we will group them based on the elements of the rare-earth group, talk about the latest developments in the biomedical applications of Gd and Eu doped CDs, and provide the recent applications of other rare-earth group metals such as Hf, Ho and other rare-earth group metals doped CDs.

### 3.1 Gadolinium

Gadolinium (Gd) is a lanthanide element with unique paramagnetism and unusually strong hydrogen proton spin lattice relaxation effects that has been widely used as MRI contrast agent and radiosensitizer [[159], [160], [161]]. Because free gadolinium ions are highly toxic, most current clinical MRI contrast agents are gadolinium chelates [162]. However, Gd^3+^ released from the complex accumulates in the body cannot be metabolized, which causes considerable biotoxicity through inhibition of calcium channels and can lead to nephrogenic systemic fibrosis in patients with renal dysfunction [163]. Therefore, there is an urgent need to find a way to improve the biosafety of gadolinium to better perform its benefits. CDs are popular for bioimaging and biosensing applications due to their unique luminescent properties and good biosafety. By doping gadolinium during the preparation of CDs, not only the problem of Gd biosafety is solved, but also the physicochemical properties of CDs are improved, which provides a novel strategy for the development of fluorescence/MRI dual-mode probes. Mauro et al. synthesized CDs-Gd by thermal decomposition using citric acid, urea and GdCl_3_. The addition of Gd^3+^ enhances MRI contrast and improves valuable properties for image-guided cancer therapy. Meanwhile, CD mitigates the toxicity of free Gd^3+^, confers fluorescence imaging features (QY = 2.1 %) and NIR photothermal conversion capabilities, allowing in vitro monitoring and photothermal therapy (PTT) applications. The experiment also demonstrated that CDs-Gd exposure to near-infrared light has different effects on healthy and cancer cells, implying that CDs-Gd kills cancer cells on demand and reduces local side effects. This multifunctional nanosystem can be used for efficient pH-dependent MRI/FLI multimodal imaging-guided photothermal therapy, offering great potential for precision cancer treatment and monitoring [164]. Li et al. used a microwave-assisted method to synthesize ultra-small-sized (∼6.42 nm) Gd-CDs. as MR/FL imaging agents with high longitudinal relaxation (12.85 mm^−1^s^−1^), intense fluorescence, excellent physiological stability and superior bio-compatibility compared to commercially used Gd-DTPA. More importantly, Gd-CDs preferentially pass through the leaky endothelium and are taken up by the ischaemic tissues of the heart without entering normal cardiomyocytes, which permits specific imaging of the infarcted area during the acute phase of MI. Gd-CDs have great potential as an efficient bimodal nanoprobe with improved reliability in the diagnosis of myocardial infarction as well as in imaging-guided surgery and therapy [165]. Table 6 summarizes the applications of Gd-doped CDs in the biomedical field.

**Table 6:** The applications of Gd-doped CDs in the biomedical field.

MCDs	Precursors	Application	λex/em	Ref.
GdNS@CQDs	GdCl_3_	Combining GdNS@CQDs with targeted ligands (folic acid, FA) and anti-cancer drugs (doxorubicin, DOX) makes it an ideal nanoplatform for in vitro fluorescence and MR dual-mode imaging and targeted drug delivery.	360/-	[166]
BCCG	L-cystein, *o*-phenylenediamine	With attractive properties such as high photothermal conversion efficiency (68.4 %), high longitudinal relaxation rate (11.84 mM), and excellent stability, it has a bright future in synergistic photothermal/photodynamic therapy (PDT) guided by fluorescence/photo-acoustic/magnetic resonance/photothermal imaging.	–	[167]
Gd-QCDs	gado-pentetic acid, Tris base	Gd-QCDs exhibits bright fluorescence, strong T1-weighted MRI contrast and low cytotoxicity.	-/445	[168]
Gd@C-dots	Gadopentetic acid	Gd@C-dots provided high r1 relaxivity (5.88 mM^−1^ s^−1^) and strong photostable fluorescence, enabling them to be used as bifunctional imaging probes to aid real-time MR imaging and immunofluorescence histology.	–	[169]
GCD	GdPM, Gd-DTPA	Gd^3+^ content containing only 1.0 % (w/w) had a high MR response with a longitudinal relaxation of 57.42 mM^−1^s^−1^ and a strong fluorescence brightness with quantum yield of 40 %.	354/445	[170]
Gd-doped CD	Gd-DTPA	Exhibits good bio-compatibility and excellent performance in terms of longitudinal relaxation rate (r1) of 6.45 mM^−1^S^−1^ and radiosensitization enhancement.	370/475	[171]
Gd-CDs	GdCl_3_, Citric acid	Gd-CDs exhibited considerable magnetic resonance properties with a longitudinal relaxation rate of 14.08 mM^−1^s^−1^ and showed very low toxicity to NCI–H446 cells.	320/446	[172]
Gd-CDs	Gd-DTPA, l-arginine	Gd-CDs showed excellent multicolor fluorescent cell labelling ability and better MR contrast effects at different excitation wavelengths, exhibiting brighter MR signals than commercial Gd-DTPA.	350/	[173]
Gd/Yb@CDs	Gadolinium chloride, ytterbium chloride	Gd/Yb@CDs Exhibited higher longitudinal relaxation rate (r1 = 6.65 mM^−1^S^−1^) and excellent X-ray absorption performance (45.43 HU L g^−1^). It can be used as a promising MRI/CT/FI multi-peak nanoprobe to provide more accurate and comprehensive diagnostic information.	340/418	[174]

### 3.2 Europium

**Fig. 8:**
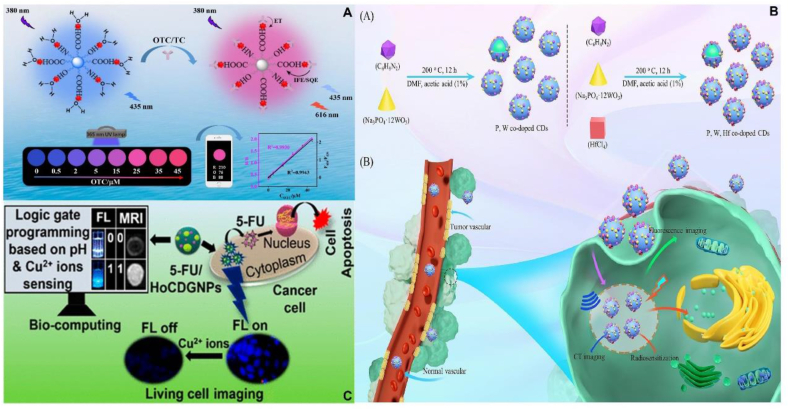
Biomedical applications of rare-earth group metal doped CDs. (A) The N-CDs-Eu^3+^ dual-response fluorescent probe has good specificity and anti-interference properties for both OTC and TC. Reprinted with permission from Wu et al. (B) P, W, Hf co-doped CDs offer significant advantages in imaging tracking and biosafety for OS. Reprinted with permission from Zhu et al. (C) HoCDGNPs provide the advantages for Cu^2+^ sensing and 5-FU pH responsive drug delivery. Reprinted with permission from Swarup et al.

Europium (Eu) exhibits strong red photoluminescence under ultraviolet radiation irradiation, and this photoluminescence can be observed not only when doped into crystal matrices or glasses, but also in europium (III) complexes with organic ligands [175]. Therefore europium (III) can be an excellent luminescent probe for biochemical or biomedical applications [176,177]. In recent years, europium-based fluorescent sensing platforms exhibit highly enhanced fluorescence when bound to tetracycline, which have attracted much attention. It has been reported that the β-diketonate conformation in tetracycline (TC) tends to coordinate violently with the europium ion (Eu^3+^) to form a stable tetracycline-Eu^3+^ complex. The transfer of energy absorbed from tetracyclines to Eu^3+^ effectively sensitizes the characteristic emission of Eu^3+^, which is also known as the antenna effect [178,179]. Based on the formation of tetracycline-Eu^3+^ complexes, which enabling the determination of TC. However, vibrations of water molecules coordinated to Eu^3+^ can cause a significant quenching effect, which results in weak emission from the tetracycline-Eu^3+^ complex and low sensitivity for tetracycline detection [180,181]. In addition, these europium-based sensor platforms are less stable in humid environments and under UV light, thus limiting them in practical applications [182]. Therefore, fluorescent sensors for Eu^3+^ are relatively unreliable and can be greatly affected by environmental conditions, instrument efficiency, and excitation factors, and it is particularly important to seek a novel sensing platform for the detection of TC based on Eu^3+^. As an emerging carbon-based nanomaterial, doping CDs with Eu^3+^ is expected to form a highly sensitive and stable sensing platform for TC detection due to its unique nanosize effect and photoluminescence properties. A dual-responsive fluorescent probe based on nitrogen-containing carbon dots (N-CDs) and Eu^3+^ heterodimers (N-CDs-Eu^3+^) was developed by Wu et al. for the selective determination of OTC and TC. N-CDs acted as a co-ligand for Eu^3+^ and as a recognition unit for OTC/TC, whereas Eu^3+^ was able to specifically recognize OTC/TC. The experimental results showed that the proportional detection ranges of OTC and TC were 0.1–45 μΜ and 0.1–30 μΜ, respectively, and the detection limits were 0.017 and 0.041 μM, respectively. The good specificity and anti-interference of this probe for both OTC and TC confirms that it has a favorable application prospect in food safety and environmental monitoring (Fig. 8A) [183]. Chen et al. successfully synthesized adenosine monophosphate/Eu (III) nanoscale coordination polymers (CD@AMP/Eu NCPs). The presence of OTC resulted in a significant fluorescence quenching of CDs due to the internal filtration effect, while the fluorescence of Eu^3+^ gradually increased due to the antenna effect of OTC. under excitation at 310 nm, the Eu^3+^ doped NCPs exhibited strong pink emission at 615 nm and blue emission at 430 nm from CDs when exposed to OTC. The ratio of fluorescence intensity (F615/F430) showed excellent linearity with a wide response range of OTC from 0.2 to 60 μ M with a lower limit of detection (LOD) of 25 nM (3σ). This work successfully developed a novel ratiometric fluorescent probe for the determination of hygromycin, providing a new solution for rapid and visual detection of OTCs [184]. In recent years, a large number of experimental studies have demonstrated the potential of europium-doped CDs as a highly sensitive and stable sensing platform for tetracycline detection [[185], [186], [187]].

### 3.3 Others

In addition to the gadolinium (Gd) and europium (Eu) elements highlighted above, hafnium (Hf), holmium (Ho) and other metals of the rare-earth group also play an important role in biomedical applications. Hafnium (Hf^3+^) is a new X-ray contrast agent that provides better X-ray absorption in the energy range of computed tomography (CT) due to its high level of electron density compared to iodine and allows images of comparable quality to be obtained at significantly lower radiation doses [[188], [189], [190]]. Zhu et al. developed P, W, Hf multi-element charge-transfer co-doped CDs as a bimodal contrast agent for osteosarcoma (OS) models. Due to the presence of valence electrons in the doped Hf^3+^, the radiative complexation of electrons and the formation of holes are promoted. P, W, Hf co-doped CDs exhibited higher bio-compatibility in Balb/C hormonal mice, good cellular imaging and fluorescence/CT high contrast imaging performance compared to undoped Hf^3+^. It also has significant advantages in terms of imaging tracking of OS and biosafety, and has good potential for use as a dual-mode imaging contrast agent (Fig. 8B) [191]. Su et al. successfully prepared a new type of hafnium-doped CDs (HfCD) from citric acid (CA), thiourea (TU) and ruthenium chloride (HfCl_4_) by a simple one-pot pyrolysis method. It was demonstrated that HfCDs could be localized at the tumor site and rapid imaging was achieved within 1 min. HfCDs have significant advantages such as stable stability, good bio-compatibility, excellent water solubility, excellent CT imaging performance, and tumor preferential accumulation ability, which gives them special features for CT/fluorescence imaging of in-situ hepatocellular carcinoma, and allows for the fabrication of excellent renal removable multimodal imaging nanoprobes with great clinical diagnostic potential [192]. Like Gd, holmium (Ho) is a paramagnetic material whose paramagnetism stems from the 4f-electrons. Furthermore, despite having a shorter spin relaxation time, Ho^3+^ has a higher magnetic moments (10.5 μB) among all lanthanide ions (even higher than Gd^3+^ (8.0 μB)) [193]. Most of the current research on Ho^3+^ has centred around their ability to enhance T2 negative contrast, and the potential of Ho^3+^ doped CDs matrices remains underexplored [194,195]. Thus, optimal FL/MR properties can be achieved by combining the potential of Ho^3+^ and CDs. Swarup et al. developed holmium doped carbon dotted gelatin nanoparticles (HoCDGNPs) for anticancer drug 5-FU delivery by a two-step desolvation method for better bimodal pH, Cu^2+^ sensing and pH response. FL quenching of HoCDGNPs formed by copper amine or copper carboxylate coordination in the presence of Cu^2+^. Furthermore, the resulting system can be used for pH-responsive drug delivery of 5-FU with sustained and selective release in the pH range 6–7.4. Fluorescence of CD in 5-FU/HoCDNPs is further used to monitor intracellular distribution and lysosomal targeting, and the advantages of assembling therapeutics, bioimaging modalities, and sensing on a single platform make it a potential contender in cancer therapeutics (Fig. 8C) [196]. Neodymium (Nd) brings interesting luminescent and magnetic characteristics to materials and the organometallic compounds. Therefore, it is a matter of interest to explore Nd-doped CDs. Aleyamma et al. synthesized a luminescent and paramagnetic Nd-doped carbon dots using *o*-phenylenediamine and NdNO_3_·6H_2_O by a one-step hydrothermal method and formed poly-CD-Nd-doped nanocomposites with poly-β-cyclodextrin. The release of loaded CPT from NCs was sustained and depended on the pH, resulting in an improved anticancer efficacy against MCF-7 cells compared to free CPT. This work demonstrated the suitability of poly-CD-Nd-doped nanocomposites as anticancer drug delivery carriers and nanomaterials traceable by luminescence/MRI imaging by studying anticancer activity against a cancer cell line (MCF-7) in vitro [197].

### 3.4 Conclusion

Rare-earth group metals play a wide and unique role in modern life. The above examples have verified that by effectively combining rare-earth metals with CDs, we have solved to a great extent the problems faced by rare-earth metals, such as poor biosafety, low utilization and weak antibacterial properties. Meanwhile, the physicochemical properties of CDs are improved, and the application fields of CDs are expanded. However, we are concerned that most of the current rare-earth doped CDs bring more value for biosensing and bioimaging applications, while little research has been done in the fields of antibacterial, cancer therapy and osteogenesis, and further in-depth studies are needed.

## 4 Summary and outlook

CDs, as a rising star in the carbon nano-family, have received extensive attention from researchers for their excellent nanoscale effects, large specific surface area, easy surface functionalization, coordinated photoluminescence, high quantum yield, and low cytotoxicity. Their small size and large specific surface area make them excellent drug carriers for drug delivery. The ease of surface functionalization allows it to provide a wide range of heteroatom doping sites to improve the physicochemical properties of the CDs. Superior optical properties make them a great advantage for bioimaging and biosensing. As better electron donors, metal ions have more unoccupied orbitals and larger atomic radius. The charge density and mode of charge transfer between the metal and CDs are changed by doping metals into CDs, which improves the physicochemical characteristics of CDs even further. Compared to undoped CDs, metal doped CDs have a higher quantum yield, superior optical characteristics, and greater potential for biological applications. In addition, metal doping onto CDs has broadened the range of applications for metals and to some extent solved the problems of limited bioavailability, instability, and poor biosafety associated with transition group and rare-earth metals. This paper describes the latest research results of transition group metal-doped CDs in recent years in the field of antibacterials, cancer therapy, osteogenesis, biosensing and bioimaging through a large number of examples, and briefly describes the latest advances in the biomedical applications of rare-earth group metals. With the gradual improvement of people's living standards and the significant increase of health awareness, the flourishing development of metal-doped CDs in the biomedical field is of great significance to the national health security in the context of the new era.

**Fig. 9:**
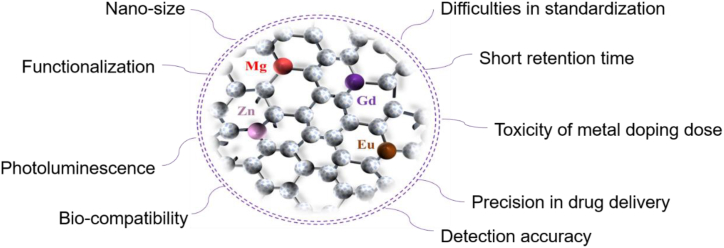
Advantages and challenges of metal-doped CDs.

Although metal-doped CDs have made encouraging progress in the biomedical field, the problems and challenges faced cannot be ignored and more efforts are needed to solve them. (Ⅰ) Standardizing preparation procedures is challenging since even after using the same approach, metal-doped CDs still exhibit variations in size and fluorescence features. Furthermore, toxicity and fluorescence characteristics are largely controlled by CD size, and the impact of metal doping on size is a significant concern. Therefore, there is still some distance to develop a biocompatible, stable and reproducible method. (Ⅱ) Due to the nanoscale effect of CDs, the retention period of CDs in the body is comparatively short, and they are easily removed from it. the action time of CDs and the transported drugs in the body are limited to a certain extent, which reduces the action effect of CDs and loaded drugs. Therefore, appropriate animal models need to be selected to carefully evaluate the duration of action of metal-doped CDs and their loaded drugs in vivo. (Ⅲ) Although metals such as Fe, Mg, Cu are essential elements for human health, the doping dose of the metals still needs our focus, so it is necessary to study the effects of metal-doped CDs on the immune system, central nervous system and reproductive system. (Ⅳ) While metal-doped CDs exhibit remarkable potential for use in cancer therapy due to their highly sensitivity to temperature and PH, accurate drug delivery to tumor cells for effective tumor death is still a hard task. (Ⅴ) Finding an improved approach with a high degree of precision to detect the target is still required since, as a fluorescent probe and imaging agent, the target material's small size or the inability to detect it at an early stage remain challenging issues. (Fig. 9).

Opportunities and challenges always go hand in hand. Each application of metal-doped CDs will benefit more from each step taken to overcome the aforementioned challenges. With the rapid advancement of science and technology, we firmly believe that metal-doped CDs will be more and more promising for biomedical applications.

## Notes

All authors have approved this version of the paper, and due care has been taken to ensure the integrity of the work. Neither the entire paper nor any part of its content has been published, accepted or submitted elsewhere. The authors declare no competing financial interest.

## Data availability statement

No data was used for the research described in the article.

## CRediT authorship contribution statement

**Jin Qi:** Writing – original draft, Validation, Methodology, Investigation. **Pengfei Zhang:** Visualization, Data curation. **Tong Zhang:** Visualization, Investigation. **Ran Zhang:** Data curation. **Qingmei Zhang:** Investigation. **Jue Wang:** Investigation. **Mingrui Zong:** Data curation. **Yajuan Gong:** Data curation. **Xiaoming Liu:** Methodology, Conceptualization. **Xiuping Wu:** Supervision, Resources, Funding acquisition. **Bing Li:** Project administration, Funding acquisition.

## Declaration of competing interest

All authors have approved this version of the paper, and due care has been taken to ensure the integrity of the work. Neither the entire paper nor any part of its content has been published, accepted or submitted elsewhere. The authors declare no competing financial interest.
